# Clinical impact of developing better practices at the institutional level on surgical outcomes after distal pancreatectomy in 1515 patients: Domestic audit of the Japanese Society of Pancreatic Surgery

**DOI:** 10.1002/ags3.12066

**Published:** 2018-03-25

**Authors:** Sohei Satoi, Tomohisa Yamamoto, Fuyuhiko Motoi, Ippei Matsumoto, Hideyuki Yoshitomi, Ryosuke Amano, Munenori Tahara, Yoshiaki Murakami, Hidehito Arimitsu, Seiko Hirono, Masayuki Sho, Hironori Ryota, Masayuki Ohtsuka, Michiaki Unno, Yoshifumi Takeyama, Hiroki Yamaue

**Affiliations:** ^1^ Department of Surgery Kansai Medical University Hirakata Japan; ^2^ Department of Surgery Tohoku University Graduate School of Medicine Sendai Japan; ^3^ Department of Surgery Faculty of Medicine Kindai University Osaka‐Sayama Japan; ^4^ Department of General Surgery Chiba University Graduate School of Medicine Chiba Japan; ^5^ Department of Surgical Oncology Osaka City University Osaka Japan; ^6^ Department of Surgery Sapporo‐Kosei Hospital Sapporo Japan; ^7^ Department of Surgery Institute of Biomedical and Health Sciences Hiroshima University Hiroshima Japan; ^8^ Division of Gastroenterological Surgery Chiba Cancer Center Hospital Chiba Japan; ^9^ Second Department of Surgery School of Medicine Wakayama Medical University Wakayama Japan; ^10^ Department of Surgery Nara Medical University Kashihara Japan

**Keywords:** distal pancreatectomy, morbidity, mortality, process of care, standardization

## Abstract

**Background and Aim:**

Institutional standardization in the perioperative management of distal pancreatectomy (DP) has not been evaluated in a multicenter setting. The aim of the present study was to assess the influence of institutional standardization on the development of postoperative complications after DP.

**Methods:**

Data were collected from 1515 patients who underwent DP in 2006, 2010, and 2014 at 53 institutions in Japan. A standardized institution (SI) was defined as one that implemented ≥6 of 11 quality initiatives according to departmental policy. There were 541 patients in the SI group and 974 in the non‐SI group. Clinical parameters were compared between groups. Risk factors for morbidity and mortality were assessed by logistic regression analysis with a mixed‐effects model.

**Results:**

Proportion of patients who underwent DP in SI increased from 16.5% in 2006 to 46.4% in 2014. The SI group experienced an improved process of care and a lower frequency of severe complications vs the non‐SI group (grade III/IV Clavien‐Dindo; 22% vs 29%, respectively, clinically relevant postoperative pancreatic fistula; 22% vs 31%, respectively, *P* < .05 for both). Duration of in‐hospital stay in the SI group was significantly shorter than that in the non‐SI group (16 [5‐183] vs 20 postoperative days [5‐204], respectively; *P* = .002). Multivariate analysis with a mixed‐effects model showed that soft pancreas, late drain removal, excess blood loss and long surgical time were risk factors for post‐DP complications (*P* < .05). Pancreatic texture, drain management and surgical factors, but not standardization of care, were associated with a lower incidence of post‐DP complications.

## 1 INTRODUCTION

Distal pancreatectomy (DP) is the standard procedure for various diseases located in the pancreas body or tail. Although high‐volume centers report low mortality rates ranging from 0% to 2%, the morbidity rate is still high, ranging from 24% to 56%.^1^, ^2^, ^3^, ^4^, ^5^, ^6^, ^7^, ^8^ The most common complication after DP is postoperative pancreatic fistula (POPF), which ranges from 0% to 61%.^1^, ^2^, ^3^, ^4^, ^5^, ^6^, ^7^, ^8^ POPF may lead to the development of severe complications, such as intra‐abdominal abscesses, delayed gastric emptying (DGE), post‐pancreatectomy hemorrhage (PPH), respiratory failure, sepsis, or death.^1^, ^2^, ^3^, ^4^, ^5^, ^6^ The surgical procedure of DP can be categorized as technically simple relative to pancreaticoduodenectomy (PD). Effective closure of the pancreatic remnant is important and remains challenging for reducing clinically relevant (CR) POPF. However, well‐defined management strategies for improving surgical outcomes are also lacking for DP.

Impact of a well‐managed process of care on clinical outcomes has been assessed in a limited, single‐institution method only, whereas the effects of standardized care on morbidity and mortality after DP have never been assessed in a multicenter setting. In the present study, we evaluated trends in clinical demographics, processes of care and postoperative complications after DP in patients in 53 Japanese institutions that participated in the Japanese Society of Pancreatic Surgery in 2006, 2010 and 2014. Next, we tested the hypothesis that the deliberate use of a process of care at an institutional level can improve morbidity and mortality after DP in relatively specialized institutions for pancreatectomy.

## 2 PATIENTS AND METHODS

The questionnaire audits consisted of two parts. The first determined institutional characteristics, and the second was the perioperative data of 1515 patients who underwent DP in 2006, 2010 and 2014 at a total of 53 institutions in the Japanese Society of Pancreatic Surgery. The audit for PD was done simultaneously and is already published.^9^

The first part of the questionnaire audit consisted of clinical questions concerning hospital volume, surgeon volume, and the 11 quality initiatives defined for the current study according to departmental policy at an institutional level, as shown in Table 1. Implementation of the quality initiatives was ranked according to levels of decision‐making authority from A to C (A, full dependence on departmental policy; B, surgeon's decision in part; C, surgeon's decision). Based on this ranking, a standardized institution was defined as one in which ≥6 of 11 quality initiatives were ranked as “A” in each year (2006, 2010 and 2014). Quality initiatives in perioperative management were determined in accordance with the presence or lack of institutional criteria for perioperative management. Hospital volume was defined as low (0‐24 PD per year), intermediate (25‐49 PD per year), and high (50 or more PD per year).^9^ Surgeon volume (number of PD/year per surgeon) was defined as low (0‐11 PD in a year) and high (12 or more PD in a year).^9^

**Table 1:** Eleven quality initiatives defined for the current study according to departmental policy at an institutional level

Surgical site infection precaution
Rehabilitation program
Pulmonary embolism prophylaxis
Duration of prophylactic antibiotic use
High‐risk patient program
Type of intraperitoneal drainage
Criteria for nasogastric tube
Criteria for drain removal
Criteria for intraperitoneal drainage
Criteria for oral intake initiation
Criteria for hospital discharge

The second part of the questionnaire audit comprised data collected from 1515 patients who underwent DP in 2006, 2010, and 2014, including patient demographics, surgical parameters, clinical outcomes, and trends examined over time. Clinical backgrounds and outcomes were compared between patients who underwent DP in standardized institutions (SI group) and in non‐standardized institutions (non‐SI group) in 2006, 2010 and 2014. Moreover, risk factors for postoperative complications and mortality were investigated. Postoperative complications were defined based on the international criteria for postoperative pancreatic fistula (POPF),^10^ delayed gastric emptying (DGE),^11^ incisional surgical site infection (SSI), and Clavien‐Dindo classification.^12^ This study was approved and overseen by the Institutional Review Board of Kansai Medical University (No. H1403101) and each participating hospital.

### 2.1 Statistical analysis

The database was investigated by biostatisticians at Statcom Co. Ltd (Tokyo, Japan), as already reported.^9^ The first questionnaire audit was common, as the data were previously reported.^9^ Continuous variables were expressed as median and range. Nominal data were compared with χ^2^ tests and continuous variables with analysis of variance. Mixed‐effect models (SAS PROC MIXED) were used to account for clustering hospitals for continuous variables. Models were constructed with manual variable selection methods. Volume and quality measures were entered manually, and additional covariates previously reported to be associated with the occurrence of postoperative complications were also selected for inclusion. Clinical impacts of standardization of perioperative management, surgeon or hospital volume, and general clinical indicators on postoperative complications were assessed by logistic regression analysis with a mixed‐effects model. *P* value <.05 was considered statistically significant. All analyses were carried out with SAS version 9.4 (SAS Institute, Inc., Cary, NC, USA).

## 3 RESULTS

### 3.1 First questionnaire audit

As reported previously,^9^ in 2014, 94% of institutions participating in this audit carried out a standardized surgical procedure of PD, 74% had a pancreatic team, 85% accrued a pancreatic database, and 90% collected surgical outcome measures. Among 53 institutions, the number of standardized institutions increased from seven in 2006 to 17 in 2010 and to 28 in 2014. Among 11 quality initiatives, in 2014, half or more of the institutions were ranked as “A” in the category of SSI precaution, rehabilitation program, pulmonary embolism prophylaxis, duration of prophylactic antibiotic use, type of biliary drainage and intraperitoneal drainage, and criteria for nasogastric tube, biliary drainage, and intraperitoneal drainage. However, the categories of high‐risk patient program, criteria for drain removal, oral intake initiation, and hospital discharge were not standardized in many institutions, even in 2014.

### 3.2 Trends of DP in 2006, 2010, and 2014

Proportion of patients who underwent DP at a SI increased from 17% in 2006 to 37% in 2010 to 46% in 2014.^9^ As shown in Table 2, number of DP carried out in these centers dramatically increased from 308 in 2006 to 515 in 2010 and to 692 in 2014. In terms of comorbidities, the proportion of patients who received anticoagulant therapy gradually increased from 6.5% in 2006 and 9.7% in 2010 to 12.1% in 2014. Although the majority of pathological diagnoses including pancreatic ductal adenocarcinoma did not change, the frequency of neuroendocrine tumors increased from 5.8% in 2006 to 10.0% in 2014.

**Table 2:** Trends in clinical parameters of patients who underwent distal pancreatectomy in 2006, 2010 and 2014

Parameter	2006	2010	2014
	n = 308	n = 515	n = 692
Background, n (%)			
Diabetes mellitus	88 (29%)	159 (31%)	204 (30%)
Liver cirrhosis	8 (2.6%)	19 (3.7%)	8 (1.2%)
Chronic obstructive pulmonary disease	8 (2.6%)	28 (5.4%)	26 (3.8%)
Chronic renal failure requiring hemodialysis	1 (0.3%)	6 (1.2%)	12 (1.7%)
Steroid use	4 (1.3%)	16 (3.1%)	16 (2.3%)
Anticoagulant therapy	20 (6.5%)	50 (9.7%)	84 (12.1%)
ASA 3‐5	19 (6.2%)	29 (5.6%)	52 (7.5%)
Pathological diagnosis			
PDAC	171 (56%)	274 (53%)	346 (50%)
Cystic disease	68 (22%)	122 (24%)	153 (22%)
Chronic pancreatitis	17 (5.5%)	10 (1.9%)	36 (5.2%)
Neuroendocrine neoplasm	18 (5.8%)	48 (9.3%)	72 (10%)
Other or unknown	34 (11%)	61 (12%)	85 (13%)
Malignancy	195 (63%)	325 (63%)	429 (62%)
NAC(R)T	3 (1.0%)	50 (9.7%)	95 (14%)
Surgical factor			
Portal vein resection, n (%)	5 (1.6%)	16 (3.1%)	20 (2.9%)
Arterial resection, n (%)	15 (4.9%)	39 (7.6%)	68 (9.9%)
Soft pancreas, n (%)	206 (67%)	373 (72%)	538 (78%)
Operative time, median (min‐max), min	270 (79‐677)	295 (89‐846)	300 (90‐780)
Extent of blood loss, median (min‐max), mL	552 (0‐6303)	400 (0‐9730)	303 (0‐10270)
Means of pancreatic transection, n (%)			
Stapler	65 (21%)	236 (46%)	368 (53%)
Scalpel	154 (50%)	177 (34%)	103 (15%)
Ultrasonic activated device	30 (10%)	51 (10%)	110 (16%)
Other	59 (19%)	51 (10%)	111 (16%)
Laparoscopic surgery	16 (5.2%)	85 (17%)	184 (27%)
Type of intraperitoneal drainage, n (%)			
Closed‐suction type	138 (45%)	307 (60%)	447 (65%)
Single‐drain use	83 (28%)	166 (32%)	330 (48%)
Days to removal (postoperative) of drain; median (min‐max)	8.0 (0‐95)	7.0 (1‐103)	5.0 (1‐154)
Blood transfusion, n (%)	55 (18%)	72 (14%)	87 (13%)
Duration of prophylactic antibiotic; median (min‐max), d	3.0 (1‐14)	3.0 (1‐11)	3.0 (1‐29)
Days to removal (post‐operative) of NG tube; median (min‐max), d	1.0 (0‐15)	1.0 (0‐57)	1.0 (0‐14)
Days to initiation (post‐operative) of oral intake; median (min‐max), d	5.0 (2‐28)	4.0 (1‐58)	4.0 (1‐53)
Postoperative complications			
Overall complications, n (%)	176 (57%)	293 (57%)	447 (65%)
In‐hospital mortality, n (%)	6 (1.9%)	6 (1.2%)	5 (0.7%)
Clavien‐Dindo grading, I‐II:IIIa/b:IVa/b:V	28:28:0.3:2.6%	33:22:1.4:1.0%	43:24:1.5:0.6%
POPF grading, A:B/C	12%:33%	18%:26%	24%:26%
DGE grading, A:B/C	2.6%:2.6%	3.1%:2.2%	2.2%:4.2%
PPH grading, A:B/C	0.6%:1.9%	1.2%:1.8%	0.6%:2.6%
Incisional SSI, n (%)	16 (5.2%)	20 (3.9%)	27 (3.9%)
Organ/space SSI, n (%)	73 (24%)	102 (20%)	133 (19%)
Readmission (within 30 d after discharge), n (%)	6 (1.9%)	20 (3.9%)	21 (3.0%)
Reoperation, n (%)	9 (2.9%)	12 (2.3%)	16 (2.3%)
Duration of in‐hospital stay; median (min‐max), d	25 (6‐122)	19 (5‐183)	17 (5‐204)

ASA, American Society of Anesthesiology; DGE, delayed gastric emptying; NAC(R)T, neoadjuvant chemo(radiation)therapy; NG, nasogastric; PDAC, pancreatic ductal adenocarcinoma; POPF, postoperative pancreatic fistula; PPH, post‐pancreatectomy hemorrhage; SSI, surgical site infection.

In terms of surgical parameters, frequency of neoadjuvant therapy, arterial resection and use of a laparoscopic approach increased over time (Table 2). Although operative time increased, extent of blood loss decreased over time. Cut and closure type of pancreatic remnant changed from the use of a scalpel to a stapler. Use of closed suction drainage systems increased, whereas that of open drainage systems decreased. Drain removal occurred at a median of 8 postoperative days (POD) in 2006, which decreased to a median of 5 POD in 2014. Although overall postoperative complication rates did not differ, in‐hospital mortality and clinically relevant (CR) POPF gradually decreased over time. Median duration of hospital stay was dramatically shortened from 25 days in 2006 to 17 days in 2014.

### 3.3 Standardized group vs non‐standardized group

Distal pancreatectomy was carried out for 541 patients in the SI group and for 974 patients in the non‐SI group. As shown in Table 3, the SI group contained a higher proportion of high‐surgeon volume centers relative to the non‐SI group (38% vs 26%, respectively; *P* < .001). In terms of drain management, a higher rate of closed suction drainage use was found in the SI group relative to the non‐SI group (71% vs 52%, respectively; *P* < .001). Moreover, the median time to drain removal in the SI group (POD‐5) was shorter than that in the non‐SI group (POD‐7, *P* < .001). In comparisons of postoperative complications, a lower incidence of overall complications (54% vs 64%), grade III/IV/V Clavien‐Dindo classification (22% vs 29%), CR‐POPF (22% vs 31%), and SSI (incisional, 2.6% vs 5.0%; organ/space, 17% vs 23%) was found in the SI group relative to the non‐SI group, respectively (*P* < .05 for all). Median duration of hospital stay in the SI group was also shorter than that in the non‐SI group (POD‐16 vs POD‐20, *P* = .002).

**Table 3:** Clinical backgrounds and outcomes: SI group vs non‐SI group

	Non‐SI (n = 974)	SI (n = 541)	*P*‐value
Case volume^a^			
Low (0‐24)	175 (18%)	122 (23%)	.091
Intermediate (25‐49)	450 (46%)	231 (43%)	
High (50+)	349 (36%)	188 (34%)	
Surgeon volume^b^			
Low (0‐11)	722 (74%)	333 (62%)	<.001
High (12+)	252 (26%)	208 (38%)	
Operative time, min	292 (79‐780)	292 (104‐846)	.287
Extent of blood loss, mL	419 (0‐10270)	343 (0‐9730)	.383
Intraperitoneal drainage, closed suction	506 (52%)	386 (71%)	<.001
Days to drain removal (POD)	7.0 (0‐154)	5.0 (1‐95)	<.001
Duration of prophylactic antibiotic	3.0 (1‐29)	3.0 (1‐13)	<.001
Days to N/G removal (POD)	1.0 (0‐51)	1.0 (0‐72)	.009
Days to initiation of oral intake (POD)	1.0 (0‐57)	1.0 (0‐28)	.204
Morbidity, n (%)	622 (64%)	294 (54%)	<.001
Mortality n (%)	3 (0.3%)	1 (0.2%)	.638
Clavien‐Dindo grading III/IV/V, n (%)	280 (29%)	117 (22%)	.002
Delayed gastric emptying, n (%)	60 (5.5%)	34 (6.2%)	.671
Clinically relevant POPF, n (%)	298 (31%)	121 (22%)	<.001
Incisional SSI, n (%)	49 (5.0%)	14 (2.6%)	.022
Organ/Space SSI, n (%)	219 (23%)	89 (17%)	.006
Readmission, n (%)	35 (3.6%)	12 (2.2%)	.139
Reoperation, n (%)	24 (2.5%)	13 (2.4%)	.932
Duration of in‐hospital stay, d	20 (5‐204)	16 (5‐183)	.002

a Case volume indicates number of PD in a year; n (%).

b Surgeon volume indicates number of PD/surgeon in a year, n (%).

Continuous variables are expressed as median (range).

N/G, nasogastric tube; PD, pancreaticoduodenectomy; POD, postoperative day; POPF, postoperative pancreatic fistula; SI, standardized institution; SSI, surgical site infection.

### 3.4 Multivariate analysis of postoperative complications

Tables 4, 5, 6, 7 show the results of multivariate logistic regression analyses to detect risk factors for each complication. Risk factors for overall complications were being a patient in a high‐hospital‐volume center and late drain removal (*P* < .05). Soft pancreas, open surgery, longer operative time and late drain removal were significantly associated with development of CR‐POPF (*P* < .05). Development of post‐pancreatectomy hemorrhage was significantly associated with the presence of vascular resection, excess blood loss, and late drain removal (*P* < .05). A significant association was found between higher American Society of Anesthesiologists (ASA) scores or presence of vascular resection and in‐hospital mortality (*P* < .05). Deliberate use of a process of care at an institutional level was not associated with improvement of morbidity and mortality after DP.

**Table 4:** Multivariate analysis with mixed‐effects model: Risk factors for overall complications

Parameter (n = 1290)		Estimate	SE	*P*‐value
Case volume	25‐49 intermediate vs 0‐24 low	−0.008	0.191	.967
	50 or more high vs 0‐24 low	0.748	0.225	<.001
Surgeon volume	12 or more high vs 0‐11 low	−0.196	0.184	.287
Standardization	SI vs non‐SI	−0.095	0.160	.554
Body mass index	≥25 vs <25	0.306	0.165	.063
Liver cirrhosis	Present vs none	−0.787	0.400	.050
ASA	3˜5 vs 1˜2	−0.127	0.253	.616
Malignancy	Yes vs no	−0.171	0.146	.241
Vascular resection	Present vs none	0.269	0.218	.218
Soft pancreas	Yes vs no	0.287	0.159	.072
Laparoscopic surgery	Yes vs no	0.312	0.190	.101
Operative time, min	≥500 vs <500	0.255	0.143	.074
Extent of blood loss, mL	≥1000 vs <1000	0.281	0.145	.052
Date of drain removal	≥6 vs <6	1.510	0.141	<.001

ASA, American Society of Anesthesiologists; SE, standard error; SI, standardized institution.

**Table 5:** Multivariate analysis with mixed‐effects model: Risk factors for clinically relevant postoperative pancreatic fistula

Parameter (n = 1286)		Estimate	SE	*P*‐value
Case volume	25‐49 intermediate vs 0‐24 low	−0.307	0.216	.154
	50 or more high vs 0‐24 low	0.142	0.256	.579
Surgeon volume	12 or more high vs 0‐11 low	−0.082	0.236	.730
Standardization	SI vs non‐SI	−0.171	0.200	.391
Body mass index	≥25 vs <25	0.303	0.171	.077
Liver cirrhosis	Present vs none	−0.354	0.441	.422
ASA	3˜5 vs 1˜2	0.251	0.276	.362
Malignancy	Yes vs no	−0.154	0.162	.341
Vascular resection	Present vs none	0.373	0.228	.102
Soft pancreas	Yes vs no	0.403	0.185	.030
Laparoscopic surgery	Yes vs no	−0.508	0.220	.021
Operative time, min	≥500 vs <500	0.574	0.158	<.001
Extent of blood loss, mL	≥1000 vs <1000	0.066	0.158	.678
Date of drain removal	≥6 vs <6	1.793	0.183	<.001

ASA, American Society of Anesthesiologists; SE, standard error; SI, standardized institution.

**Table 6:** Multivariate analysis with mixed‐effects model: Risk factors for postoperative pancreatic hemorrhage

Parameter (n = 1289)		Estimate	SE	*P*‐value
Case volume	25‐49 intermediate vs 0‐24 low	0.404	0.544	.458
	50 or more high vs 0‐24 low	0.651	0.591	.271
Surgeon volume	12 or more high vs 0‐11 low	0.157	0.469	.738
Standardization	SI vs non‐SI	−0.427	0.433	.324
Body mass index	≥25 vs <25	0.143	0.398	.719
Liver cirrhosis	Present vs none	−0.188	1.084	.862
ASA	3˜5 vs 1˜2	−0.167	0.647	.797
Malignancy	Yes vs no	0.475	0.432	.272
Vascular resection	Present vs none	1.083	0.431	.012
Soft pancreas	Yes vs no	−0.252	0.383	.511
Laparoscopic surgery	Yes vs no	0.298	0.558	.594
Operative time, min	≥500 vs <500	−0.333	0.366	.362
Extent of blood loss, mL	≥1000 vs <1000	1.063	0.398	.008
Date of drain removal	≥6 vs <6	1.008	0.427	.019

ASA, American Society of Anesthesiologists; SE, standard error; SI, standardized institution.

**Table 7:** Multivariate analysis with mixed‐effects model: Risk factors for in‐hospital mortality

Parameter (n = 1293)		Estimate	SE	*P*‐value
Case volume	25‐49 intermediate vs 0‐24 low	−0.760	1.090	.486
	50 or more high vs 0‐24 low	−0.568	1.243	.648
Surgeon volume	12 or more high vs 0‐11 low	0.701	1.107	.527
Standardization	SI vs non‐SI	1.415	0.911	.121
Body mass index	≥25 vs <25	1.043	0.756	.168
Liver cirrhosis	Present vs none	1.013	1.376	.462
ASA	3˜5 vs 1˜2	3.135	0.857	<.001
Malignancy	Yes vs no	0.745	1.171	.525
Vascular resection	Present vs none	2.552	0.863	.003
Soft pancreas	Yes vs no	−0.104	0.816	.899
Operative time, min	≥500 vs <500	0.485	0.835	.561
Days of drain removal	≥6 vs <6	1.724	0.977	.078

ASA, American Society of Anesthesiologists; SE, standard error; SI, standardized institution.

## 4 DISCUSSION

The present study evaluated trends in the clinical practice of DP over time, and the effect of streamlining and standardizing processes of care at the institutional level on patient outcomes using the data of 1515 patients from 53 relatively specialized institutions for pancreatectomy in Japan, which is one of the biggest cohorts. As expected, our data showed that the number of SI has increased, and early drain removal, use of closed suction drainage and early hospital discharge were achieved more frequently over time. Laparoscopic approaches and DP with arterial resection, such as DP with celiac axis resection, have been more frequently carried out in a wider patient population, including patients with anticoagulant medication, and a longer operative time was needed, but the extent of blood loss decreased dramatically over time. Moreover, the SI group had the standardized process of care in terms of use of closed suction drainage, a higher proportion of early drain removal, and a shorter duration of antimicrobial therapy. In addition, the SI group was associated with lower rates of overall complications, severe complications (Clavien‐Dindo III‐V), CR‐POPF, and SSI. However, multivariate analyses with a mixed‐effects model showed that the SI group did not have a lower incidence of postoperative complications. Development of CR‐POPF was significantly associated with pancreas texture, type of surgery, operative time and time to drain removal. In particular, time to drain removal was one of the risk factors for overall complications, CR‐POPF, and PPH, with overall complications and CR‐POPF. Presence of vascular resection was closely associated with the development of PPH and in‐hospital mortality.

Several authors have reported that high‐volume and specialized centers achieve better surgical outcomes after pancreatectomy.^13^, ^14^, ^15^ However, Riall et al suggested that there is still significant variability in the outcomes of pancreatic resection.^16^ Lucas and Pawlik have proposed that quality improvement efforts should focus not only on who is operating or where the operation occurs (surgeon or hospital volume), but also on how the process occurs.^17^

Which measures beyond morbidity and mortality may better reflect quality in DP? These measures include traditional clinical outcomes, as well as processes of care and structural elements of care. Among them, the “process of care” can be under the control of surgeons and the medical staff. Vollmer et al proposed that improved process management can mitigate the impact of preoperative risk and effectively deliver quality advances, despite traditional outcomes that may already meet or exceed benchmark outcomes for a given major surgical procedure.^18^ Implementation of a clinical pathway as a tool for introducing a well‐established process of care has been reported to be associated favorably with short‐term outcomes after DP, including length of hospital stay in single institutional studies.^19^, ^20^, ^21^, ^22^

Recently, we reported that the standardized adoption of a well‐organized process of care for PD at the institutional level, but not hospital/surgeon volumes, was associated with a reduction in post‐PD complications in a multicenter setting.^9^ PD is a complicated surgery which consists of multi‐organ resection with at least three anastomoses, and it is associated with high morbidity and mortality. Therefore, standardization of the surgical technique and perioperative management is greatly required, and can be a critical indicator for assessing the clinical outcomes of PD. In contrast, the surgical procedure of DP can be categorized as technically simple relative to PD. Perioperative management of patients who undergo DP is also simple in terms of the absence of pancreatico‐enteric anastomosis. In this study, standardization of the perioperative care process at the institutional level did not affect the occurrence of post‐DP complications including CR‐POPF. The international multi‐institutional distal pancreatectomy study group analyzed data from 2026 patients who underwent DP. Although they failed to predict CR‐POPF occurrence reliably, seven risk factors (age, body mass index [BMI], serum albumin level, pathology, epidural use, splenectomy, and vascular resection) were identified.^23^ They suggested the existence of two possibilities: (i) fistula after distal pancreatectomy is a stochastic process that cannot be predicted; or (ii) despite the extensive data accrual by each collaborating institution, important risk factors were not accounted for. Unlike PD, risk factors for post‐DP complications seem to have diversity. In the present study, a standardized institution was defined as one in which ≥6 of 11 quality initiatives (as shown in Table 1) were managed according to full dependence on departmental policy. Among them, the criteria of drain removal and hospital discharge and a high‐risk patient program had not been standardized in half or more institutions (data not shown). In this study, the occurrence of overall complications, CR‐POPF, and PPH were closely related with late drain removal. In fact, several articles have reported that unnecessarily prolonged drainage might itself increase postoperative morbidities such as CR‐POPF and infectious complications.^20^, ^24^, ^25^ The spread of an early drain removal policy, even in post‐DP management, may reduce postoperative complications.

The present study has some potential limitations. First, although we attempted to include all measures of process of care in each institution and to use the definition of SI consistently, other important indicators or methods for assessing “standardization” might exist. Second, we assessed the fact that a conscious attempt was made to improve the process of care in each institution, but we could not evaluate that the actual processes were applied more frequently or more regularly. Thus, perioperative management strategies varied across institutions. However, this provides a realistic picture, reflecting inherent variability in the clinical practice of DP. Third, institutions participating in this study are specialized centers for pancreatectomy (or include at least one surgeon certified by the Japanese Society of Hepatobiliary Pancreatic Surgery) and, therefore, the findings may not be generalizable to all hospitals.

## 5 CONCLUSIONS

Standardized adoption of a well‐organized process of care for DP at the institutional level did not reduce post‐DP complications. Traditional factors such as pancreatic texture, drain management and surgical factors were associated with a lower incidence of post‐DP complications. Sustainable efforts will be required to reduce post‐DP complications.

## DISCLOSURE

Funding: This study was financially supported by the Japanese Society for Clinical Pathway.

Conflicts of Interest: Dr Satoi was supported by a grant from the Japanese Society of Clinical Pathway. Professor Unno is supported by grants from the Taiho, Takeda, Chugai, Novartis, Astellas, Yakult, and Toyama Chemical Pharmaceutical Companies. Other authors declare no conflicts of interest for this article.

Author Contributions: SS and TY contributed to all aspects of this study and article. FM, IM, HY, RA, MT, YM, HA, SH, MS, HR, MO, MU, YT, and HY contributed to study conception, collection of data and critical revision of the article. All authors approved the final draft of the article.
