# Supplementary material for: Clinical impact of developing better practices at the institutional level on surgical outcomes after distal pancreatectomy in 1515 patients: Domestic audit of the Japanese Society of Pancreatic Surgery
Source: Ann Gastroenterol Surg. 2018 Mar 25;2(3):212–9. doi: 10.1002/ags3.12066 (PMC5980579; doi:10.1002/ags3.12066)
Supplement: Supplementary file 1 [file AGS3-2-212-s001.docx]

Supplemental Table. Participated institutions

1. Department of General Surgery, Chiba University Graduate School of Medicine
2. Department of Surgery, Tohoku University Graduate School of Medicine
3. Second Department of Surgery, School of Medicine, Wakayama Medical University
4. Department of Surgery, Kansai Medical University
5. Department of Gastroenterological Surgery (Surgery II), Nagoya University Graduate School of Medicine
6. Department of Surgery, Teikyo University School of Medicine
7. Division of Gastroenterological Surgery, Chiba Cancer Center Hospital
8. Department of Surgery, Nara Medical University
9. Department of Surgery, Kindai University Faculty of Medicine
10. Department of Gastroenterological Surgery II, Hokkaido University Graduate School of Medicine
11. Division of Clinical Medical Science, Department of Surgery, Hiroshima University Graduate School of Biomedical Sciences
12. Department of Surgical Oncology, Osaka City University Graduate School of Medicine
13. Department of Surgery, Sapporo Kosei Hospital
14. Department of Surgery, Jichi Medical University
15. Departments of Surgery, Hyogo College of Medicine
16. Department of Surgery, Hiroshima City Hiroshima Citizens Hospital
17. Department of Hepatobiliary Pancreatic and Transplant Surgery, Mie University Graduate School of Medicine
18. Department of Hepato-Biliary-Pancreatic Surgery, Kobe University Graduate School of Medicine
19. Department of Surgical Oncology and Gastroenterological Surgery, Sapporo Medical University School of Medicine
20. Department of Surgery, Kitasato University
21. Department of Surgery, Tokyo Metropolitan Cancer and Infectious Diseases Center Komagome Hospital
22. Department of Surgery, School of Medicine, Kurume University
23. Department of Surgery Nagasaki University Graduate School of Biomedical Sciences
24. First Department of Surgery, Yamanashi University School of Medicine
25. Department of Gastroenterological and General Surgery, St. Marianna University School of Medicine
26. Department of Surgery, Keio University School of Medicine
27. Department of Surgery, National Hospital Organization Osaka National Hospital
28. Department of Biliary Pancreatic Surgery, Fujita Health University School of Medicine
29. Department of General and Gastroenterological Surgery, Osaka Medical College
30. Department of Surgery, Kyorin University
31. Department of Hepato-Biliary-Pancreatic Surgery, Osaka City General Hospital
32. Department of Digestive Surgery, Kyoto Prefectural University of Medicine
33. Department of Gastroenterological Surgery, Graduate School of Medical Science, Kanazawa University
34. Department of Surgery, Matsuyama Red Cross Hospital
35. Department of Surgery, Fukuyama City Hospital
36. Department of Gastroenterological Surgery, Kagawa University
37. Department of Surgery, Tenri Hospital
38. Department of Gastroenterological Surgery, Yokohama City University School of Medicine
39. Department of Digestive Surgery and Surgical Oncology, Yamaguchi University Graduate School of Medicine
40. Department of Surgery, Niigata Prefectural Central Hospital
41. Department of Surgery, National Hospital Organization Nagasaki Medical Center
42. Department of Surgical Oncology, Kagoshima University
43. Department of Surgery, Toho University Ohashi Medical Center
44. Department of Surgical Oncology, Gifu University, Graduate School of Medicine
45. Department of Surgery, Nakagami Hospital
46. Department of HBP and Transplantation Surgery, Ehime University Graduate School of Medicine
47. Department of Surgery, Tokyo Dental College Ichikawa General Hospital
48. Hokkaido University Graduate School of Medicine Gastroenterological Surgery I
49. Department of Surgery and Science, Graduate School of Medical Science, Kyushu University
50. Department of Surgery, Tokyo Medical University Ibaraki Medical Center
51. Department of Surgery, Tobata General Hospital
52. Departments of Surgery, Tochigi Cancer Center
53. Department of Surgery, Nippon Medical School
